# Structure of ring-shaped A*β*_42_ oligomers determined by conformational selection

**DOI:** 10.1038/srep21429

**Published:** 2016-02-12

**Authors:** Linh Tran, Nathalie Basdevant, Chantal Prévost, Tâp Ha-Duong

**Affiliations:** 1 BIOCIS, Univ. Paris-Sud, CNRS, Université Paris-Saclay, Châtenay-Malabry, 92290, France; 2 LAMBE, CNRS, Université Evry-Val-d’Essonne, Evry, 91025, France; 3 LBT, Univ. Paris-Diderot, CNRS, Sorbonne Paris-Cité, Paris, 75005 France

## Abstract

The oligomerization of amyloid beta (A*β*) peptides into soluble non-fibrillar species plays a critical role in the pathogenesis of Alzheimer’s disease. However, it has been challenging to characterize the tertiary and quaternary structures of A*β* peptides due to their disordered nature and high aggregation propensity. In this work, replica exchange molecular dynamics simulations were used to explore the conformational space of A*β*_42_ monomer. Among the most populated transient states, we identified a particular conformation which was able to generate ring-shaped pentamers and hexamers, when docked onto itself. The structures of these aggregates were stable during microsecond all-atom MD simulations in explicit solvent. In addition to high resolution models of these oligomers, this study provides support for the conformational selection mechanism of A*β* peptide self-assembly.

Many studies about the Alzheimer’s disease (AD) physiopathology suggest that the *in vivo* presence of insoluble fibrillar plaques of amyloid-*β* peptides weakly contributes to cognitive impairement and neurodegeneration^1^,^2^,^3^. In constrast, the level of soluble non-fibrillar A*β* aggregates, with molecular mass ranging from about 10 to 100 kDa^4^, was shown to strongly correlate with synaptic dysfunction and neuron loss severity^2^,^5^,^6^. Several classes of soluble A*β* oligomers were identified, with different size, morphology and toxicity, depending on the experimental conditions^7^,^8^,^9^,^10^,^11^,^12^.

Among them, A*β*_42_ pentamers/hexamers, which can assemble into decamers/dodecamers, were emphasized by several groups as the primary toxic species in AD. Toxic hexamers of synthetic A*β* were first identified at low temperature and named “ADDL” (A*β*-Derived Diffusible Ligands) by the Klein’s group^13^. They reported later that physiological temperatures favor the formation of dodecamers, which were also detected in solutions prepared from brains of AD patients^14^. In parallel, Barghorn *et al*. were able to prepare a homogenous solution of stable dodecamers, using small amounts of sodium dodecyl sulfate (SDS)^15^. They demonstrated that this species, named “globulomer”, also exists *in vivo* and induces synapse dysfunction, suggesting that ADDL and globulomer could be similar assemblies^10^. The latter, however, seem to differ from the dodecamer, named “A*β* * 56”, produced by middle-aged transgenic mice which express a human amyloid-*β* precursor protein (APP) variant^7^,^10^. Indeed, A*β* * 56 level remains stable between 6 and 15 months of age and transiently impairs spatial memory without permanent neurodegeneration, whereas ADDL and globulomer levels continuously increase during the same period, inducing learning deficit and neuronal death^9^,^12^.

One of the earliest structural information on A*β*_42_ soluble pentamers/hexamers was provided by Bitan *et al*. by a combination of several biophysical methods^16^. Using photo-induced cross-linking of unmodified proteins (PICUP), these authors observed A*β*_42_ pentamer or hexamer units, named “paranuclei”, with a quasi-circular structure of about 5 nm in diameter, and which were further able to self-assemble into larger non-fibrillar oligomers^16^. In addition, it was emphasized that the A*β* two hydrophobic residues Ile41 and Ala42 play an essential role in paranuclei formation^16^,^17^. Later on, using ion mobility coupled with mass spectroscopy (IM/MS) and measurements of cross-sections, Bernstein *et al*. demonstrated that A*β*_42_ paranuclei have a planar hexagonal geometry and can stack with each other into dodecamers^18^. In parallel, using notably high resolution atomic-force microscopy (AFM) and NMR experiments, Ahmed *et al*. observed and characterized disc-shaped A*β*_42_ pentamers/hexamers of about 10 nm in diameter and 2 nm in height which can also stack with each other to form decamers/dodecamers^19^. These assemblies are composed of loosely packed A*β*_42_ peptides characterized by a turn-strand-turn-strand-turn-strand motif, the turns being located at position 13–15, 25–29 and 37–38, bringing residues Phe19 and Leu34 in contact^19^. Moreover, these authors showed, using NMR measurements of hydrogen-deuterium exchange of the backbone amide protons, that the peptide N-termini are solvent-accessible up to the residue Gly9, whereas their C-termini are protected from the solvent, indicating that they are oriented toward the center of the discs. All together, these observations suggest that these disc-shaped oligomers represent similar species as paranuclei.

Despite this information, high resolution structural data are still necessary to clarify whether the identified pentamers/hexamers represent the same or distinct species and to study the conformational pathways between these assemblies. In this regard, Lendel *et al*. recently reported the solid-state NMR-derived structure of a hexamer of an A*β*_42_ variant in which a disulfide bond was introduced between two cysteines replacing residues Ala21 and Ala30^20^. Their hexameric assembly has a barrel-like structure of about 3 nm in diameter and height. However, the question whether the wild-type A*β*_42_ ADDL, globulomer, A*β* * 56 or paranuclei assemblies ressemble this structure remains to be elucidated.

In that context, molecular modeling can propose hypothesis on A*β* oligomer structures and provide clues helping this investigation. Several theoretical works simulated the formation and/or the stability of A*β* oligomers, either using the NMR structure of A*β*_17–42_ fibril^21^ as initial conformations^22^,^23^,^24^,^25^,^26^,^27^, or starting from random ensembles of isolated monomers^26^,^28^,^29^,^30^,^31^,^32^. However, none of these studies revealed or emphasized oligomeric ring-shaped structures, to the best of our knowledge. One of the rare MD simulations of cyclic oligomers even showed that a disc-shaped pentamer, arranged like the structure observed by Ahmed *et al*.^19^, quickly disassociates into random loop-like monomers^33^, challenging the simulation of stable ring-shaped A*β* pentamers/hexamers.

To provide atomic level models of A*β*_42_ pentamers/hexamers, we used an alternative approach based on the “conformational selection” paradigm for molecular recognition and self-organization^34^,^35^,^36^. This one postulates that any protein adopts an ensemble of different conformations, and a molecule which may bind to it selects the most favorable conformation. In our case, A*β*_42_ peptide is well known to dynamically visit various conformations, including random coiled, helical and extended structures. These latter would be selected by another peptide in a similar conformation for binding and self-aggregation. Within this framework, our strategy to model A*β*_42_ pentamer/hexamer structures consisted in three steps. We first sampled the A*β*_42_ peptide conformational space using replica exchange molecular dynamics (REMD) simulations. This first step was locally extended around a particularly interesting conformation with a standard MD simulation. Then we selected the conformation that best fits the available experimental data on the peptide structure within pentamers/hexamers, and performed coarse-grained docking calculations of the selected conformation on itself to generate possible oligomer structures. These latter were finally submitted to microsecond MD simulations in order to assess their stability.

## Results

### A*β*_42_ conformational ensemble

**Figure 1:**
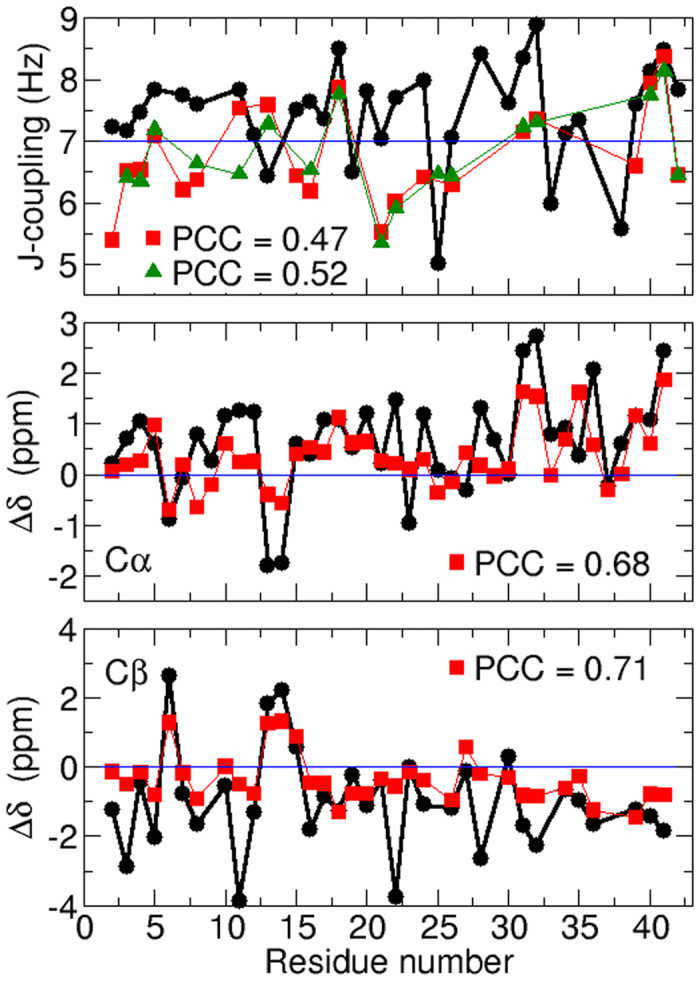
Top: Comparison of simulated 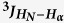-coupling constants (black circle) with experimental measurements published in 2007 (red square)^37^ and 2008 (green triangle)^38^. J-couplings are calculated from the backbone *ϕ* and *ψ* dihedrals using the Vuister and Bax parameters^77^. Middle and bottom: Comparison of theoretical C*α* and C*β* secondary chemical shifts (black lines) with experimental measurements (red lines)^43^. Secondary chemical shifts are calculated as Δ*δ* = *δ*_*coil*_ − *δ* where *δ*_*coil*_ values are taken from Wishart *et al*.^80^.

The ensemble-averaged 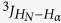-coupling constants of the A*β*_42_ residues are shown in Fig. 1 and compared with NMR measurements available in the literature^37^,^38^. Overall, the theoretical J-coupling values are in mediocre agreement with the NMR measurements, the Pearson correlation coefficients (PCC) between calculations and experiments being about 0.5. This result indicates that only a limited portion of the peptide conformational space was explored by our REMD simulations. Comparatively, the N, C*α* and C*β* chemical shifts calculated over the A*β*_42_ conformational space are in a very good agreement with NMR data (Fig. S1), the PCC values ranging from 0.90 to 0.99. However, as emphasized by in-depth studies^39^,^40^, chemical shifts can poorly discriminate between different conformational spaces, and secondary chemical shifts should be used instead to validate simulations. Comparison of the A*β*_42_ ensemble-averaged C*α* and C*β* secondary chemical shifts with NMR measurements (Fig. 1) exhibits a good agreement, with noted PCC values about 0.7. All together, these results indicate that, although not fully explored, a significant part of the A*β*_42_ conformational ensemble was sampled by the REMD simulations.

Over this explored part of the A*β*_42_ conformational space, most of the residue 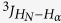-coupling constants and C*α*/C*β* secondary chemical shifts are in the range of 6–8 Hz and close to 0 ppm, respectively. This indicates that monomeric A*β*_42_ peptide is mainly in a disordered random coil structure, in line with most of the experiments. Nevertheless, our REMD simulations revealed several residues having persistent secondary structures, indicated by 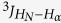-couplings outside the range of 6–8 Hz and C*α*/C*β* secondary chemical shifts greater than 1–2 ppm (in absolute value). For instance, the residues His6 and His13-His14 significantly visited helical conformations, as evidenced by their far negative C*α* and positive C*β* secondary chemical shifts (Fig. 1). Conversely, transient *β*-strand structures can be detected for hydrophobic residues Arg5, Val18, Ile31-Ile32, Val36, Val39-Val40-Ile41, in agreement with NMR measurements. Other residues visited extended conformations in our simulation (Glu3, Glu11, Glu22, Lys28), but NMR data cannot confirm these tendencies. These latter residues being all charged, this indicates that secondary structures at these positions should be strongly sensitive to the ionic and/or pH conditions. Interestingly, the A*β*_42_ C-terminal residues Val39-Val40-Ile41, which have a clear propensity to form a *β*-strand in both simulations and experiments, are often emphasized for their important role in A*β* aggregation through *β*-strand/*β*-strand interactions.

**Figure 2:**
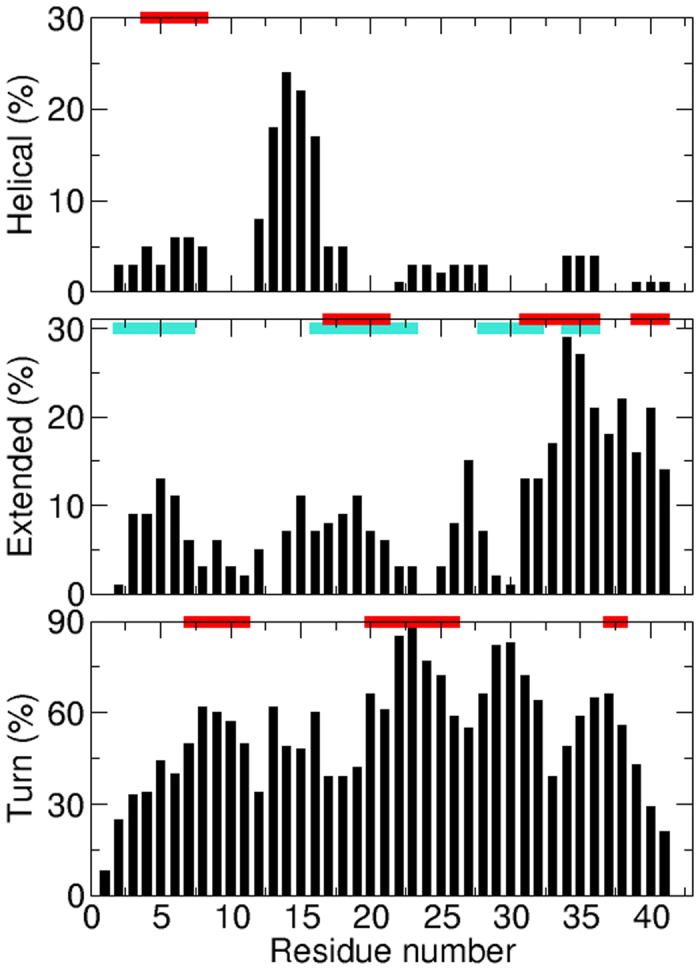
Ensemble-averaged percentage of helical (top), extended (middle) and turn (bottom) conformations as a function of the A*β*_42_ residues. Secondary structures were assigned using the STRIDE program^41^,^42^. The horizontal red bars indicate the locations of helical, extended and turn structures from NMR experiments by Hou *et al*.^44^ and those in cyan by Wälti *et al*.^43^.

The propensity of A*β*_42_ residues to form secondary structures is specified in Fig. 2 which displays the ensemble-averaged percentage of helical, extended and turn conformations, calculated with STRIDE^41^,^42^. We noted that several segments of A*β*_42_ transiently adopt secondary structures: Helical conformations can be observed at residues 4–8 and 12–16. The C-terminal region 31–41 has a significant propensity to form *β*-strands. We also found residual extended structures for segments 3–7, 14–21 and 26–28. Lastly, A*β*_42_ residues 8–9, 22–23, 29–30 and 36–37 have high propensity to form turns. Overall, these findings are in good agreement with conclusions from several NMR experiments: A*β*_42_ peptide is intrinsically disordered, with a slight tendency toward helix at position 4–8, with residual *β*-strand structure for segments 2–7, 16–23, 28–36 and 39–41, and turn conformations at residues 7–11, 20–26 and 37–38 (Fig. 2)^43^,^44^. Compared to other theoretical studies^45^,^46^, our results are comparable to those generated by Mitternacht *et al*. using a Monte Carlo approach with effective potentials and implicit solvent^47^. They are also similar to those obtained by Yang and Teplow with the AMBER force field and Generalized Born solvent model^48^. And they are very close to those reported by Rosenman *et al*. who used the OPLS-AA force field and TIP3P water^49^. This indicates that our explored conformational ensemble largely overlaps with theirs and contributes to provide a convergent picture of the A*β*_42_ transient secondary structures.

### Structural analysis of the most populated states

**Figure 3:**
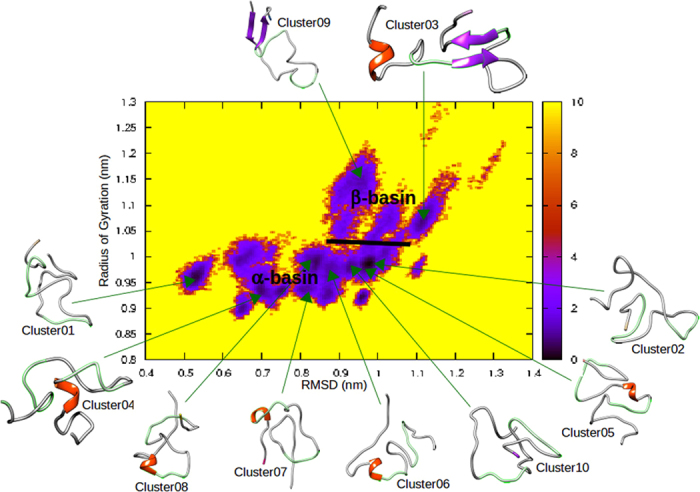
Free energy (kcal/mol) surface of A*β*_42_ peptide as a function of its RMSD relative to the initial random coil and its radius of gyration.

To gain insight into the A*β*_42_ metastable states, a reduced free energy surface was calculated as a function of its RMSD relative to the initial random coil and its radius of gyration. It reveals about twenty energy minima associated to the peptide metastable conformations (dark stain in Fig. 3). These minima are separated by energy barriers that can be only of 1–2 kcal/mol, indicating that conformational conversions can occur frequently. The free energy surface can be divided into two sub-areas, the first one covering compact conformations with radius of gyration below 1.05 nm, the other one covering extended structures with radius of gyration between 1.05 to 1.20 nm. This observation is in line with the radius of gyration distribution found by Ball *et al*., which exhibits a high and narrow peak around 1 nm and a smaller and broader one around 1.3 nm (excepting their few very extended conformations with radius of gyration up to 3 nm)^40^.

The A*β*_42_ conformations sampled during the ten lowest temperature trajectories were clustered according to their structural similarity using a RMSD threshold of 0.2 nm. Over a total of 194 generated clusters, the ten most populated ones were structurally characterized (Table S1) and a representative conformation of each cluster was located in the peptide free energy surface (Fig. 3). This analysis shows that the compact basin mainly encompasses random coiled structures with residual helical segments, while the extended one covers conformations rich in *β*-strands. This result is very similar to the partition of the A*β*_42_ conformational space into an “*α*-basin” and a “*β*-basin” reported by Yang and Teplow^48^. It is also in agreement with several circular dichroism (CD) studies which monitored random coil/*α* to *β* conformational conversions of the A*β*_42_ peptide as a function of time^50^, fraction of water^51^ or temperature^52^.

The *β*-basin in the reduced free energy surface is divided into two distinct sub-basins, one represented by the conformation cluster03 and the other by cluster09 (Fig. 3). Both structures have two *β*-strands arranged in an antiparallel *β*-sheet. The N-terminal region 3–6 laterally connects the C-terminal residues 35–38 in cluster09, whereas the segment 19–21 hydrogen bonds the region 33–35 in cluster03. Strikingly, this latter presents several structural features in common with structures derived from NMR experiments on isolated monomers^38^,^43^,^44^, but also with conformations of protomers within soluble oligomers^19^,^20^ (Table 1). (As a reminder, a protomer denotes a monomeric peptide within an oligomer). These conformations exhibit a residual (in monomers) or persistent (in oligomers) turn-*β*-turn-*β*-turn motif at very similar positions, characterized by contacts between residue Phe19 and Leu34/Met35 and a salt-bridge between Asp23 and Lys28 side chains which is important for A*β* aggregation^53^,^54^,^55^. The turn motif at position 37–38 also enables the extended C-terminal residues 39–41 to come close to the *β*-strand formed by residues 34–36. It could be noted that this turn-*β*-turn-*β*-turn motif was not retrieved in the less populated clusters generated by the REMD simulations. All together, these remarks support the hypothesis of the “conformational selection” mechanism for A*β* oligomerization and suggest that cluster03 could be one “selected” intermediary in the pathway to soluble oligomers. Nevertheless, in contrast to NMR observations, the Asp23-Lys28 salt-bridge was not seen in cluster03. This prompted us to further explore the A*β*_42_ conformational space around the latter and assess its stability.

### Molecular dynamics of cluster03

**Figure 4:**
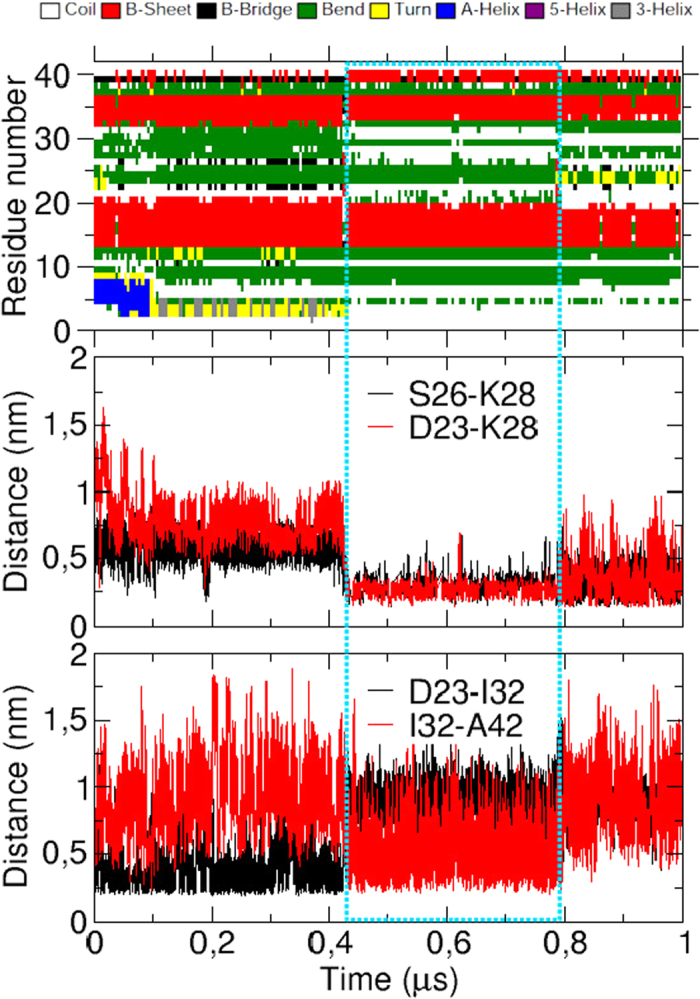
Time evolution of cluster03 secondary structures (top) and minimal distances between the side chains of selected residues. The cyan dotted box indicates the transient conformation cluster03b in which Asp23 and Lys28 form a salt-bridge.

**Figure 5:**
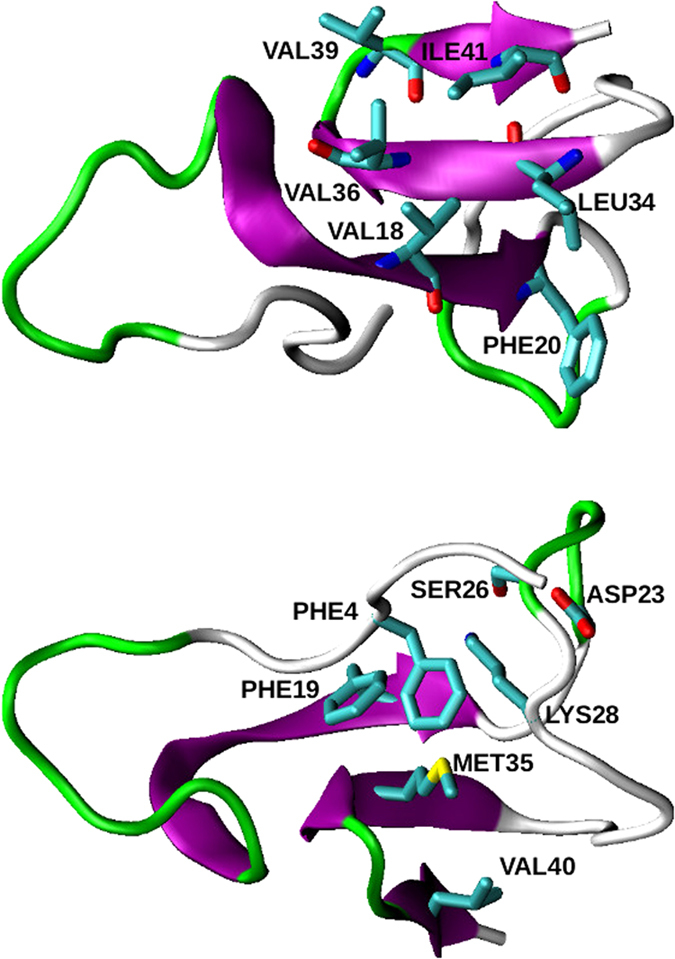
Central structure of the conformational ensemble sampled between 430 and 790 ns during the additional MD simulation of cluster03. Top and bottom view of the *β*-sheet (purple) are respectively displayed on top and bottom. Green ribbons represent the turn motifs.

For this purpose, we performed an additional 1 *μ*s MD simulation starting from cluster03, at constant pressure *P* = 1 atm and constant temperature *T* = 310 K. The time evolution of the peptide secondary structures is displayed in Fig. 4. Overall, the cluster03 conformation is quite s over the simulation duration, especially the two *β*-strands at regions 14–20 and 34–36. Nevertheless, the peptide undergoes two noticeable conformational changes. The first one, occuring around 100 ns, is the unwinding of the *α*-helix 4–8 into a coiled tail. This result is in line with NMR experiments which detected this helix with measurements of H*α* chemical shifts, but not with NOE or NH exchange data, indicating a weakly stable helix at this location^44^. This unwinding is favored by the ionic force between Glu3 and Lys28 and the hydrophobic interaction between Phe4 and Lys28, which pull and bring the peptide N-terminus in contact to the turn 23–30 (Fig. 5).

The second structural evolution, occuring between 430 and 790 ns, is the stabilization of a third *β*-strand at the C-terminal segment 39–41, which forms hydrogen bonds with the *β*-strand at positions 34–36 (Fig. 5). This observation is in line with several experimental studies which detected this transient C-terminal *β*-strand, from H*α* and C*β* chemical shifts^44^, fluorescence of A*β*-GFP fusions^56^, ^15^N spin relaxation^57^ or CD monitoring^58^. These studies also emphasized the important role of this C-terminal *β*-strand in the aggregation property of A*β*_42_. Interestingly, the stabilization of the *β*-strand at position 39–41 seems to be correlated to the bridging of the Lys28 *ε*-amino group with the side chains of Asp23 and Ser26 (Fig. 4). This relationship can be explained as follows: During the first 430 ns of the MD simulation, the Asp23 carboxylic group points toward the solvent and its methylenic group is in contact with Ile32 side chain (Fig. 4). When the Asp23 side chain binds the Lys28 one, it separates from Ile32, which comes close to the Ala42 side chain (Fig. 4). This new hydrophobic contact reinforces the lateral interactions between the two extended segments 32–36 and 39–42.

In summary, the additional MD simulation, starting from cluster03, revealed a metastable conformation, hereafter referred to as cluster03b, which has the same morphology as the former, but with a N-terminus in coiled conformation instead of being helical, a salt-bridge between Asp23 and Lys28 which sligthly rearranges the turn 23–30 conformation, and a stiffened C-terminus stabilized in *β*-strand. Since these structural features were emphasized in both monomeric and oligomeric A*β*_42_ peptides (Table 1), cluster03b is thought to be the “selected” conformation that will self-aggregate to form soluble ring-shaped paranuclei. For these reasons, cluster03b was thereafter used to run rigid docking calculations to build these oligomers.

### Structure of A*β*_42_ pentamer and hexamer

**Figure 6:**
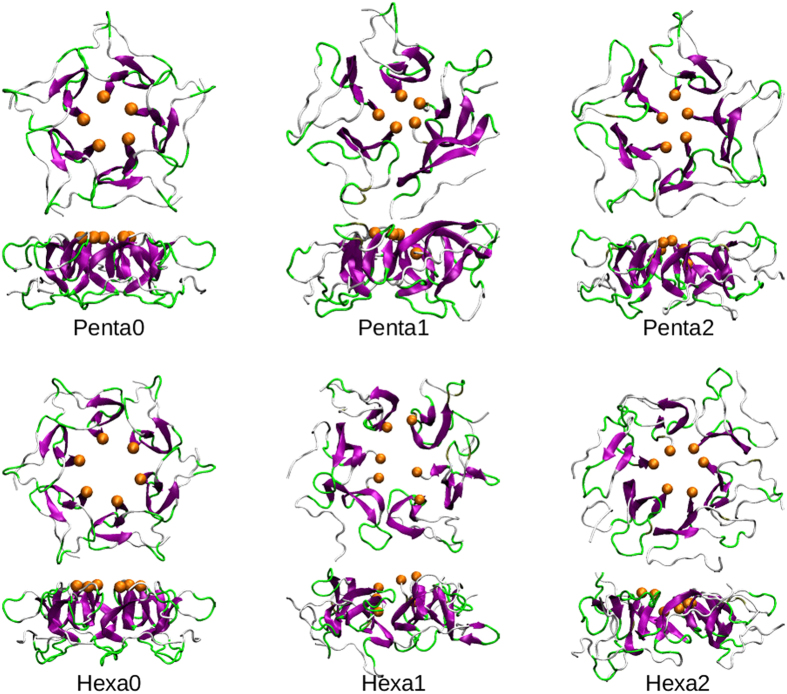
Top and side views of A*β*_42_ oligomers generated by Heligeom^59^ (Penta0 and Hexa0) and by MD simulations (Penta1, Penta2, Hexa1 and Hexa2). The protomer *β*-strands and turns are colored in purple and green, respectively. The C-terminal residues Ala42 are represented in an orange sphere.

The docking of cluster03b against itself generated 20 899 dimeric conformations, from which Heligeom can generate helical polymers of A*β*_42_ by repeating the peptide-peptide interface using screw transformations^59^. Among these, 139 assemblies were nearly circular with a helical pitch lower than 2 nm and a number of protomers per turn between 5 and 6, and 17 of them have their C-terminus oriented towards the ring center. The lowest energy oligomer meeting these criteria is a near cyclic helix with 5.2 protomers per turn and a pitch of 1.1 nm. From the latter, Heligeom generated a ring-shaped pentamer and hexamer (denoted Penta0 and Hexa0) (Fig. 6), by adjusting the protomer-protomer interfaces with a Monte Carlo optimization^59^. The diameters of these oligomers are equal to 5.1 and 5.8 nm for the pentamer and hexamer, respectively. Both oligomers thickness is about 2.1 nm. Their inside pore has a diameter of 1.2 and 1.5 nm for the pentamer and hexamer, respectively. In both oligomeric structures, the C-terminal *β*-strand of each protomer is located toward the ring center and approximatively perpenticular to the ring plane. The turn motifs are mostly located on the outward solvent exposed surface of the oligomers.

**Figure 7:**
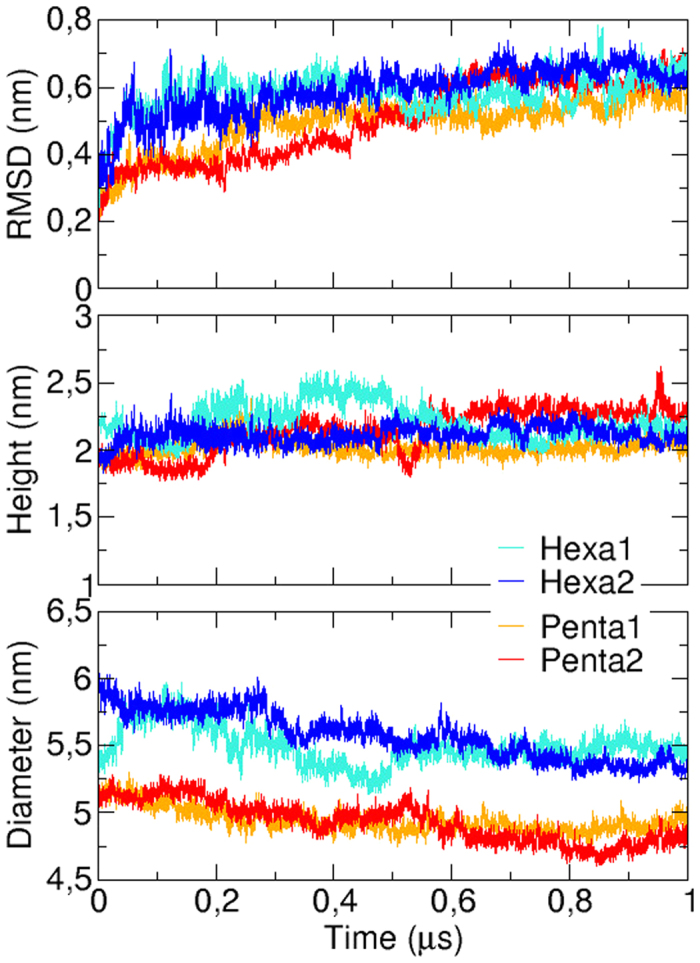
Time evolution of the pentamer (orange and red lines) and hexamer (cyan and blue lines) RMSD relative to their initial conformation (top), height (middle) and diameter (bottom).

Each model of A*β*_42_ oligomers was submitted to two independent all-atom MD simulations of 1 *μ*s in length to assess their stability. The time evolution of their RMSD relative to the initial conformation (Fig. 7) shows that the four trajectories (referred to as Penta1, Penta2, Hexa1 and Hexa2) congerved toward equilibrated conformations after about 600 ns. Representative conformations of the most populated clusters issued from these simulations (Fig. 6) illustrate the conservation of their ring-shaped morphology. One can note that, while the height of simulated oligomers remained quite constant around 2.1 nm (Fig. 7), their diameter slightly decreased toward about 4.8 and 5.3 nm for the pentamers and hexamers, respectively, indicating more compact assemblies than the initial ring-shaped structures.

**Figure 8:**
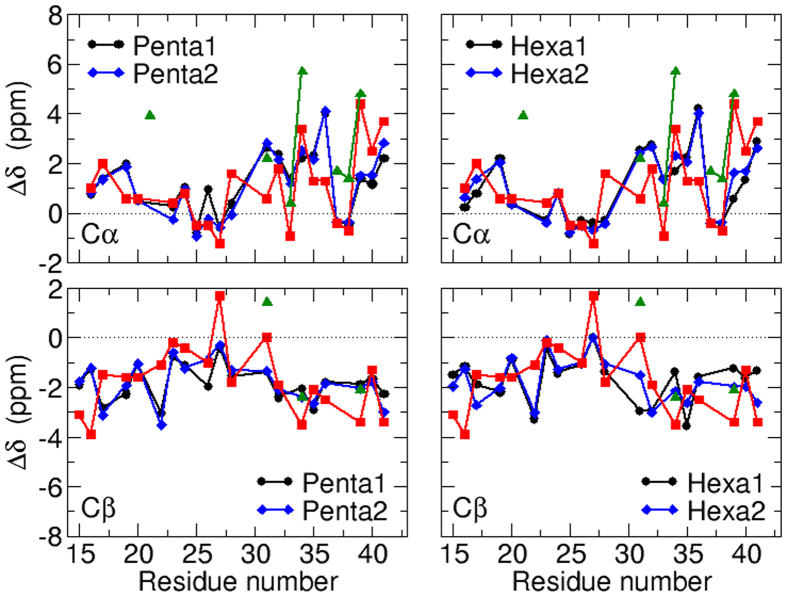
Comparison of the C*α* (top) and C*β* (bottom) secondary chemical shifts of A*β*_42_ peptides within our oligomer models, within the hexamer from Lendel *et al*.^20^(red squares) and within the pentamer from Ahmed ***et al***.^19^ (green triangle).

The time evolution of the secondary structures for each protomer within the Penta1, Penta2, Hexa1 and Hexa2 assemblies is illustrated in Figs S2 and S3, respectively. Overall, the three *β*-strands of A*β*_42_ peptide within the four oligomers are well conserved during the microsecond trajectories. Nevertheless, the *β*-strand in region 14–20 can be partially lost in some protomers, like in the fourth one of Penta1, the third one of Penta2, or in the second and fourth ones of Hexa2. Conversely, one can observe some extensions of the *β*-strands at positions 14–20 and 34–36, like in the first protomer of Penta1 or in the first, third and fourth ones of Hexa1. This induces a slight tilt of the involved peptide turn-*β*-turn-*β*-turn motif with respect to the axis perpendicular to the ring plane, but no increase of the oligomers height. When averaging over the MD trajectories and over the five or six protomers of each oligomer, the secondary chemical shifts of the A*β*_42_ peptide within Penta1, Penta2, Hexa1 and Hexa2 are in good agreement with those measured on a hexameric peptide barrel by Lendel *et al*.^20^ (Fig. 8). The agreement is also acceptable when comparing with measurements on the loosely packed pentamers by Ahmed *et al*.^19^, particularly in the peptide C-terminal region. This indicates that A*β*_42_ protomers within our simulated oligomers kept similar secondary structures to those within NMR-derived pentameric and hexameric assemblies.

**Figure 9:**
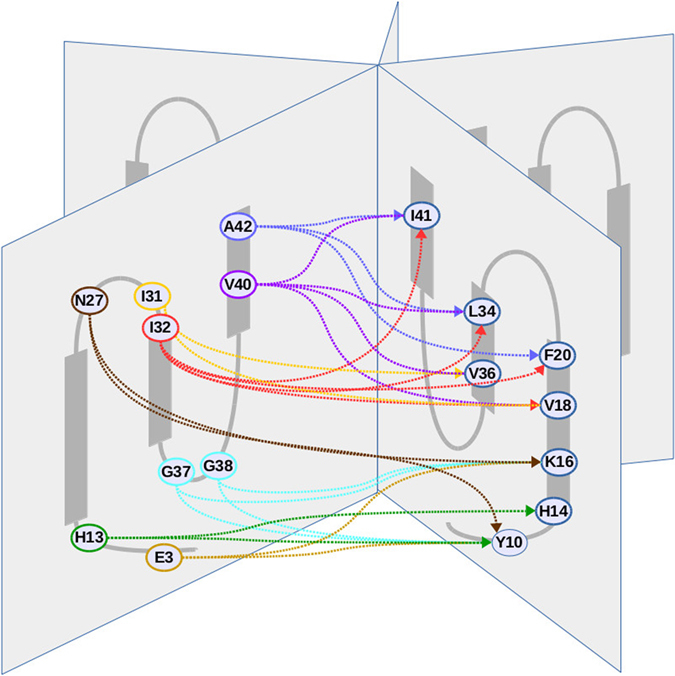
Schematic representation of A*β*_42_ pentamer highlighting the main residues of one protomer which make contacts with neighbouring protomer residues. It should be noted that in the simulated oligomers, the average plane of the three *β*-strands are slightly tilted relative to the axis perpendicular to the ring plane, and that the turns are not coplanar with these *β*-strands.

The contacts between residues of two neighbouring protomers are shown in Figs S4 and S5. Pentamer and hexamer contact maps exhibit similar patterns, in which we can identify four A*β*_42_ regions (the N-terminus 1–5, the turn 12–14, the turn 27–32 and the *β*-strand 39–42) making close contacts with four other segments (the tail 6–10 and the three *β*-strands 14–20, 34–36 and 39–42) on the neighbouring protomer (Fig. 9). Mainly, the apolar residues located on one side of the C-terminal *β*-hairpin (residues Ala42, Val40, Ile32 and Ile31) make hydrophobic contacts with the apolar residues located on the other side of the three *β*-strands of the neighbouring protomer (residues Ile41, Val36, Leu34, Phe20 and Val18). The hydrophobic cluster composed of the C-terminal residues Val40, Ile41, and Ala42 observed at the protomer-protomer interface provides a rational for the fact that A*β*_42_ is more prone to form paranuclei species than A*β*_40_. On the outward surface of the oligomers, the ring-shaped structure is maintained by intermolecular contacts between residues Gly38, Gly37, Asn27, His13 and Glu3 of one protomer and residues Lys16, His14 and Tyr10 of the neighbouring peptide. These polar interactions might be related to the strong dependency of A*β*_42_ oligomer structures on the ionic and/or pH conditions used in experiments.

## Discussion

The pentameric and hexameric oligomers of A*β*_42_ peptide were built on the basis of our cluster03b structure which has a similar morphology as the protomer conformation within the oligomers identified at low temperature by Ahmed *et al*.^19^. These structures are characterized by a turn-*β*-turn-*β*-turn-*β* motif at very similar positions (Table 1). The percentage of *β*-strand residues is about 43% which is close to the 44% value measured by Fourier transform infrared spectroscopy (FTIR) of oligomers^19^. Nevertheless, in our model, this motif is notably maintained by intramolecular hydrophobic interactions of Phe20 with Leu34 and Phe19 with Met35, whereas Ahmed *et al*. emphasized a contact between the Phe19 and Leu34 side chains^19^. This difference in the packing of side chains results in a more planar *β*-sheet in our model than in the NMR structure of A*β*_42_ oligomers. This difference in the “flatness” of the three *β*-strands could in turn explain the difference in compactness and diameter between the experimentally observed oligomers and our models. Indeed, the pentameric and hexameric models have a diameter of about 4.8 and 5.3 nm, respectively, whereas the oligomers observed by transmission electron microscopy (TEM) and AFM consists in loosely packed monomers in a disc-shape of diameter between 10 and 15 nm^19^. In our oligomer models, the average plane of the three *β*-strands tends to stack with each other. This stacking would be less compact if the *β*-sheet is less planar. Regarding the height of our ring-shaped oligomers (2.1 nm for both pentamers and hexamers), it is in good agreement with the one measured by AFM and TEM.

Our A*β*_42_ hexamer models also exhibit some strong similarities with the hexameric peptide barrel recently resolved by Lendel *et al*., using solid-state NMR spectroscopy and molecular modeling studies of a variant of A*β*_42_, in which a disulfide bond was introduced between two cysteines replacing the two residues Ala21 and Ala30^20^. First, the conformation of the A*β*_42*CC*_ protomers within their oligomer model is also characterized by a *β*-turn-*β*-turn-*β* motif, but the first and second *β*-strands (regions 15–22 and 26–36, respectively) are more extended than in our models (segments 14–20 and 34–37, respectively), in which the region Glu22-Gly33 forms a compact loop that is folded back toward the first *β*-hairpin of each monomer. Secondly, the interface between two neighbouring protomers also consists in contacts between the hydrophobic residues of one face of the *β*-sheet and the hydrophobic residues of the other face of the adjacent protomers. Nevertheless, due to the more extended region Glu22-Gly33 of the A*β*_42*CC*_ variant, its hexamer presents a loop region extended from a compact core region composed of the central and C-terminal residues. This extended loop region which is not observed in our models results in a hexamer height of 3 nm.

Ring-shaped hexamers of A*β*_42_ peptide were also detected by Bernstein *et al*. using ion mobility spectrometry^18^. In addition to A*β*_42_ dimers, tetramers and hexamers, their study detected dodecamers consisting in two stacked hexamers, but no 18-mers. Similarly, using AFM and TEM, Ahmed *et al*. observed dimers of disc-shaped pentamers or hexamers (decamers or dodecamers) with twice the height of the pentamers and hexamers^19^. We propose here plausible quaternary structures of such decamers and dodecamers, by performing (with our coarse-grained approach implemented in PTools) single docking calculations of the A*β*_42_ cyclic pentamer and hexamer models onto themselves, respectively. We found that the thirteen and twelve lowest energy predicted structures of the pentamer-pentamer and hexamer-hexamer assemblies, respectively, consisted in two rings stacked through their faces composed of the peptide N-terminal regions (Fig. S6). These results indicate a preferential mode of binding for the ring-shaped oligomers through only one of their two faces. This could account for the absence of 18-mers in the ion mobility spectrometry studies^18^ and suggests that the decameric and dodecameric paranuclei are terminal species that cannot form higher order assemblies.

In conclusion, our study supports the “conformational selection” mechanism that governs the oligomerization of A*β* peptides. Within this framework, we provided structural models at the atomic level of ring-shaped A*β* pentamers and hexamers, which were experimentally identified but only characterized at a low resolution. Our approach consisted in three steps. First, among the conformational ensemble of A*β*_42_ monomer, explored by REMD simulations and locally extended with a standard MD simulation, we selected a specific structure which has the same morphology as the protomer within pentamers and hexamers determined by NMR experiments. Then we performed docking calculations of this conformation against itself to generate dimers. Some of the generated lowest energy dimers were able to generate near cyclic pentamers and hexamers by repeating the protomer-protomer interface. The ring-shaped pentamer and hexamer having the peptide C-terminus oriented toward the center were selected and were shown to be stable during microsecond MD simulations. Docking calculations of ring-shaped pentamer and hexamer against themselves generated among the lowest energy complexes decameric and dodecameric species composed of two rings stacked through only one of their faces. Our analyses suggest that the various oligomers observed by different groups and named globulomer or A*β* * 56 are likely the same dodecameric species formed from the stacking of two hexameric paranuclei.

## Methods

### REMD simulation

We explored the conformational ensemble of A*β*_42_ peptide using the REMD technique^60^ implemented in the GROMACS molecular modeling package^61^,^62^. Simulations were performed at the all-atom level with the OPLS-AA force field^63^ in explicit solvent using the SPC/E water model^64^. The OPLS-AA force field was chosen because previous studies showed that it generated A*β*_42_ conformations in good agreement with NMR data^37^ and is suitable for simulating the aggregation of several A*β* fragments^65^. Although these previous studies used the TIP3P water model, SPC/E was recently shown to generate structures of the fragment A*β*_21–30_ similar to those obtained with TIP3P, when using OPLS-AA^66^. The crystal structure 1Z0Q^51^ was taken from the Protein Data Bank as a starting conformation. However, this structure was solved in a micellar solution and is mainly helical. It was then pre-equilibrated in vacuum using a 10 ns MD simulation at 300 K in order to generate a random coiled conformation as the initial structure for equilibration and production. The peptide was solvated with 4734 water molecules and its charge was neutralized with three sodium ions. The REMD simulations consist in MD simulations of 48 replicas of the system at temperatures ranging from 290 K to 425 K. Temperatures were fixed using the “temperature generator for REMD-simulations” webserver developped by Patriksson and van der Spoel^67^, for an exchange probability of 20%. The 48 replicas were equilibrated at constant temperature and pressure (*P* = 1 atm), using first the Berendsen coupling methods^68^ for 2 ns (*τ*_*t*_ = 0.1 and *τ*_*p*_ = 0.5 ps) and then the Nose-Hoover/Parrinello-Rahman algorithms^69^,^70^,^71^ for an additional 2 ns (*τ*_*t*_ = 0.5 and *τ*_*p*_ = 2.5 ps). The non-bonded interactions were treated using the smooth PME method^72^ for the electrostatic terms and a cutoff distance of 1.2 nm for the van des Waals potentials. The covalent bond lengths were kept constant using the LINCS^73^ and SETTLE^74^ procedures. A leap-frog algorithm was used to integrate the equations of motion with a time step of 2 fs. Exchanges between replicas were attempted every 20 ps. Each of the 48 simulations was run for 100 ns.

### Structural analysis

Similarly to the approach used in the studies of A*β* monomers by Sgourakis *et al*.^37^,^75^, we chose to analyze the ten lowest temperature trajectories, representing a cumulative simulation time of 1 *μ*s, in order to improve the statistics of the peptide sampled conformations. These temperatures, which are 290.00, 292.45, 294.93, 297.42, 299.92, 302.44, 304.98, 307.53, 310.10 and 312.69 K, are distributed around the temperature of 300 K, which can be considered as the temperature at which the “numerical experiment” was conducted. The A*β*_42_ conformational ensemble generated by REMD was compared to the experimental one as evidenced by NMR spectroscopy. First, the ensemble-averaged backbone geometry of A*β*_42_ was characterized by the 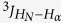-coupling constants as a function of its sequence. They were calculated from the dihedral angles *ϕ* and *ψ* using the Karplus equation^76^ and the Vuister and Bax empirical parameters^77^. The calculated 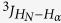-coupling constants were compared to two sets of NMR measurements, the first one being read from Fig. 1 of the article published by Sgourakis *et al*. in 2007^37^, the second one being taken from Fig. 2 of the Supporting Information of the study by Yan *et al*. in 2008^38^. Both NMR measurements of the peptide J-couplings were performed at 273.3 K. Secondly, we compared the C*α* and C*β* secondary chemical shifts of the modeled A*β* peptide with NMR data. The theoretical chemical shifts were calculated using the SHIFTS program^78^,^79^, and were subtracted to the random coil values from Wishart *et al*.^80^ to provide the secondary chemical shift values. The A*β*_42_ experimental chemical shifts were taken from Table S1 of the Supporting Information of the article by Wälti *et al*., who performed the NMR measurements at 277.15 K^43^. It should be noted that our results extracted from trajectories generated at temperatures around 300 K, cannot strictly be compared with NMR data which were collected at low temperatures (273.3 and 277.15 K). However, the A*β* monomer conformations can hardly be characterized by experiments at room temperature, due to its high aggregation propensity, and only experimental data collected at low temperature were available in the literature to assess the extent of our exploration of the peptide conformational space. The A*β*_42_ peptide secondary structures were analyzed using the STRIDE program^41^,^42^. The secondary structure characteristics were grouped into three classes: helical (type H, G or I), extended (E or B) and turn (T).

To provide a comprehensible overview of the A*β*_42_ metastable states, a reduced free energy surface was calculated as a function of the monomer RMSD relative to the initial random coil conformation and its radius of gyration. Free energies were evaluated using the equation 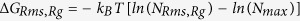, where *N*_*Rms*,*Rg*_ is the number of structures with a RMSD and a radius of gyration values around *Rms* and *Rg*. In parallel, the conformations sampled during the ten lowest temperature trajectories were clustered according to their structure similarity. The gromos method^81^ implemented in the GROMACS g_cluster tool was used with a RMSD cut-off of 0.2 nm. The first ten cluster conformations were then located in the previous reduced free energy surface.

### Coarse-grained protein-protein docking and oligomer building

Structures of A*β*_42_ dimers were generated using the molecular modeling library PTools^82^. This toolbox enables to perform protein-protein docking calculation at a coarse-grained level and its new release can now handle various force fields. We used here the SCORPION coarse-grained force field which was designed to study protein-protein recognition^83^,^84^. SCORPION was able to successfully reproduce the quaternary structure of several protein-protein complexes, starting from the bound conformation of the two partners, by using an energy minimization procedure without any bias. PTools performs systematic rigid-body docking, starting from initial regular positions and orientations of the ligand around the receptor surface, at a distance slightly larger than its largest dimension. The docking procedure consists in minimizing the interaction energy between the two partners, using the ligand six translational and rotational degrees of freedom. The minimized complex conformations were finally clustered by similarity and ranked according to their interaction energies.

The lowest energy structures of A*β*_42_ dimers, obtained by docking, were analyzed regarding the polymeric assemblies that can be generated if the protomer-protomer interface was repeated, using the Heligeom module in PTools^59^. More specifically, using screw transformations, Heligeom generates helical polymers of proteins from the structure of a dimer (composed of a protomer1 and a protomer2) by adding a protomer3, in such a way that the protomer2-protomer3 interface is identical to the one between protomer1 and protomer2, and so on. Among the provided results, we selected the helical polymers with between 5 and 6 protomers per turn and nearly cyclic with a pitch lower than 2 nm. From these selected assemblies, Heligeom is then able to generate ring-shaped pentamers and hexamers of A*β*_42_, by an adjustment procedure, using an automated Monte Carlo energy-minimization, during which cyclic geometry is enforced^59^. Finally, to check their stability, the coarse-grained oligomers were converted into atomic representations and then submitted to microsecond all-atom MD simulations, in the NPT ensemble (*T* = 310 K and *P* = 1 atm) using the same parameters as for the REMD simulations.

## Additional Information

**How to cite this article**: Tran, L. *et al*. Structure of ring-shaped A*β*_42_ oligomers determined by conformational selection. *Sci. Rep.* **6**, 21429; doi: 10.1038/srep21429 (2016).

## Supplementary Material

Supplementary Information

## Figures and Tables

**Table 1:** Comparison of the residual secondary structure positions and intramolecular residue proximity in monomeric A*β*_42_ peptide (Mono1^44^ and Mono2^43^), within soluble oligomers (Oligo1^19^ and Oligo2^20^), and in cluster03 (from REMD sampling) and cluster03b (from additional MD) conformations.

	**Mono1**	**Mono2**	**Oligo1**	**Oligo2**	**cluster03**	**cluster03b**
	**Residual secondary structure positions**					
turn	7–11		13–15		9–13	7–13
*β*-strand	17–21	16–23	17–21	16–22	14–21	14–20
turn	20–26		25–29	23–28	23–30	23–28
*β*-strand	31–36	28–36	31–36	28–36	33–36	34–36
turn	37–38		37–38	37–38	37–38	37–38
*β*-strand	39–41			39–41		39–41
	Intramolecular residues proximity					
19–(34/35)	×		×		×	×
23–28	×		×			×
(34/35/36)–(39/40/41)	×			×	×	×
