# Supplementary material for: Structure of ring-shaped Aβ42 oligomers determined by conformational selection
Source: Sci Rep. 2016 Feb 12;6:21429. doi: 10.1038/srep21429 (PMC4751476; doi:10.1038/srep21429)
Supplement: Supplementary Information [file srep21429-s1.pdf]

SUPPORTING INFORMATION FOR
Structure of ring-shaped Aβ_{42} oligomers determined by conformational selection

Linh Tran[1], Nathalie Basdevant[2], Chantal Prévost[3], and Tâp Ha-Duong[1,*]

[1]BIOCIS, Univ. Paris-Sud, CNRS, Université Paris-Saclay, Châtenay-Malabry, 92290, FRANCE
[2]LAMBE, CNRS, Université Evry-Val-d'Essonne, Evry, 91025, FRANCE
[3]LBT, Univ. Paris-Diderot, CNRS, Sorbonne Paris-Cité, Paris, 75005 FRANCE
[*]tap.ha-duong@u-psud.fr

ABSTRACT

The oligomerization of amyloid beta (Aβ) peptides into soluble non-fibrillar species plays a critical role in the pathogenesis of Alzheimer's disease. However, it has been challenging to characterize the tertiary and quaternary structures of Aβ peptides due to their disordered nature and high aggregation propensity. In this work, replica exchange molecular dynamics simulations were used to explore the conformational space of Aβ_{42} monomer. Among the most populated transient states, we identified a particular conformation which was able to generate ring-shaped pentamers and hexamers, when docked onto itself. The structure of these aggregates were stable during microsecond all-atom MD simulations in explicit solvent. In addition to high resolution models of these oligomers, this study provides support for the conformational selection mechanism of Aβ peptide self-assembly.

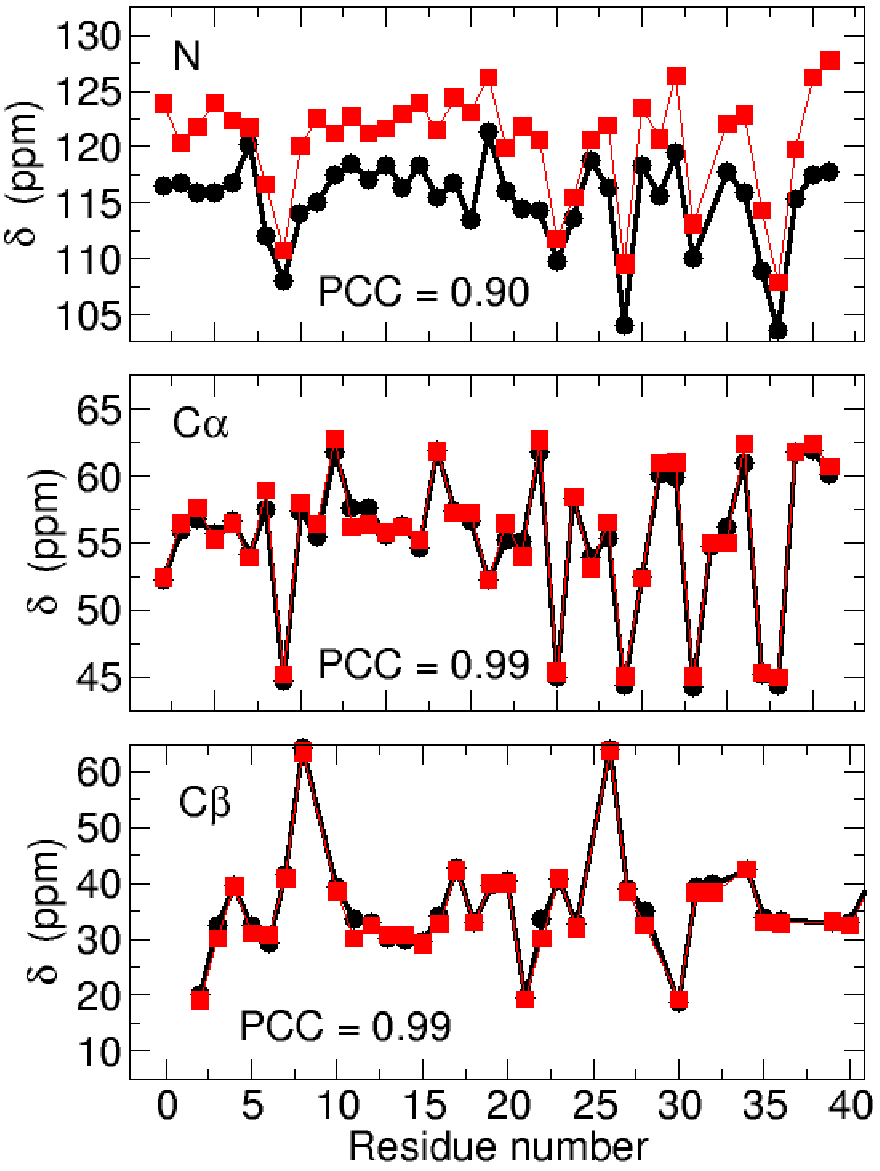

Figure S1. Comparison of theoretical N (top), Cα (middle) and Cβ (bottom) chemical shifts (black lines) with experimental measurements (red lines).[1]

Cluster	Population (%)	RMSD (nm)	Gyration (nm)	N^o of α-residues	N^o of β-residues
01	9.4	0.54	0.97	0	0
02	9.3	0.98	0.99	0	0
03	5.5	1.11	1.06	4	14
04	4.7	0.71	0.94	3	2
05	4.0	0.97	0.98	3	4
06	3.7	0.88	0.96	4	4
07	3.5	0.83	0.94	3	2
08	3.4	0.84	0.99	4	2
09	3.4	0.95	1.16	0	9
10	2.9	0.93	0.97	0	2

Table S1. Structural characteristics of the first ten clusters of the Aβ_{42} conformations. The RMSD is calculated relative to the initial random coil.

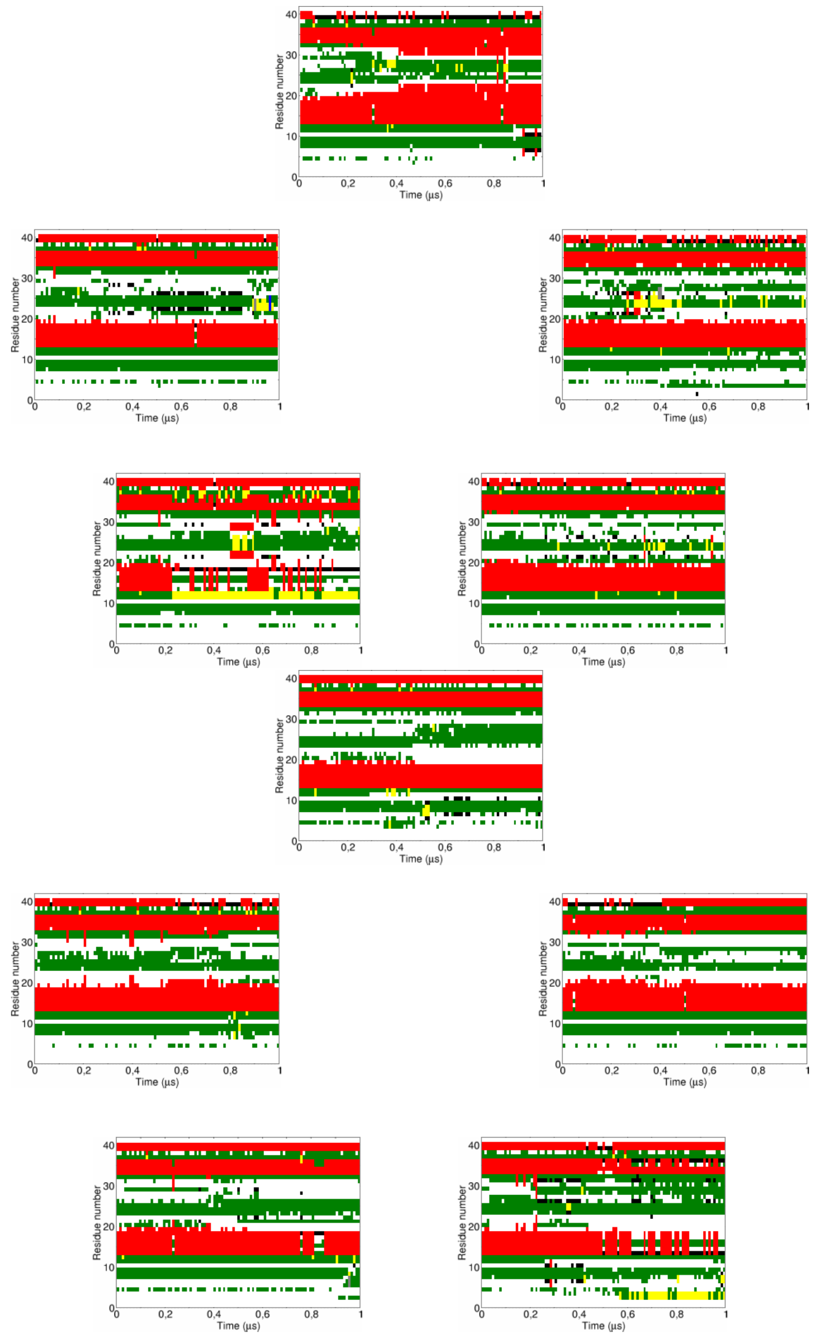

Figure S2. Time evolution of the secondary structures for the five Aβ_{42} peptides within the Penta1 (top) and Penta2 (bottom) oligomers.

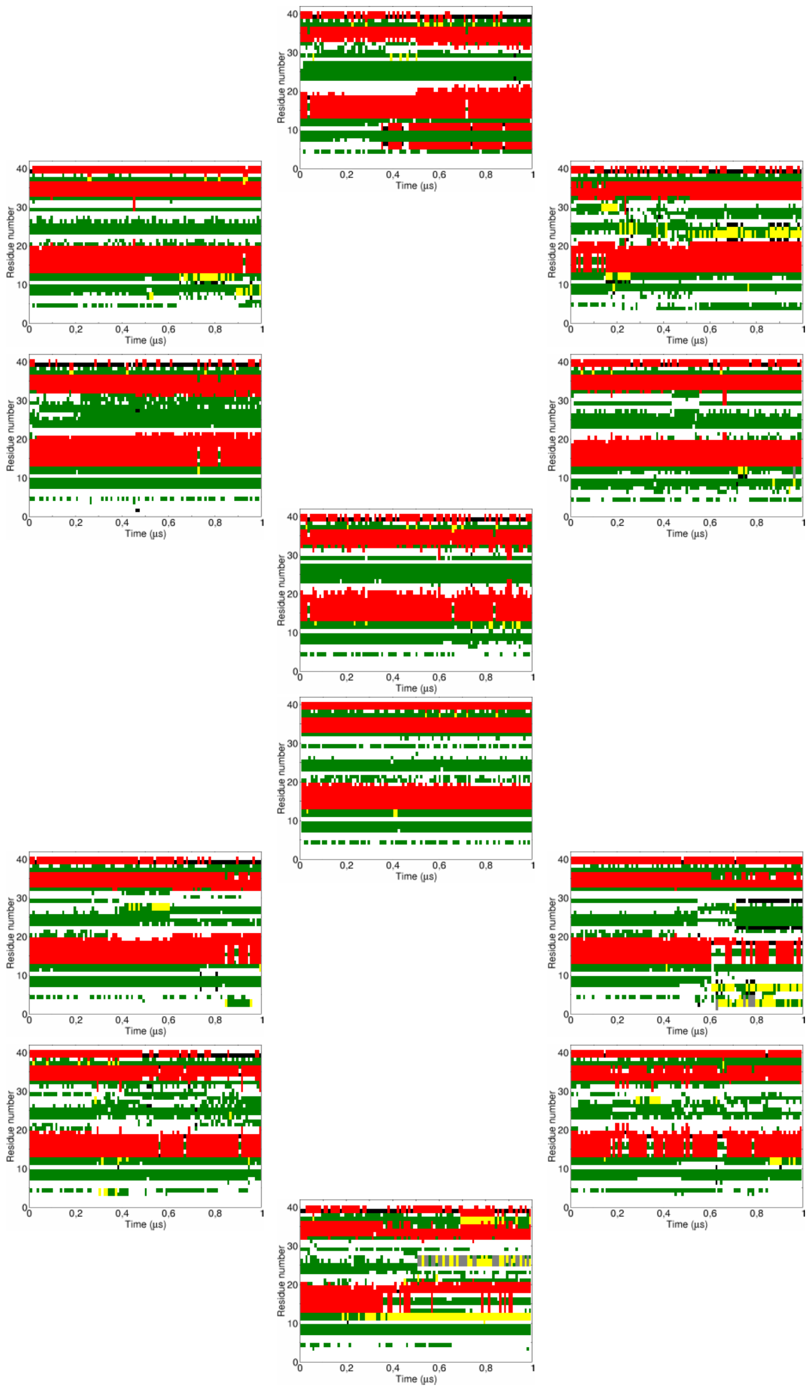

Figure S3. Same as Fig. S2 but for the Hexa1 (top) and Hexa2 (bottom) oligomers.

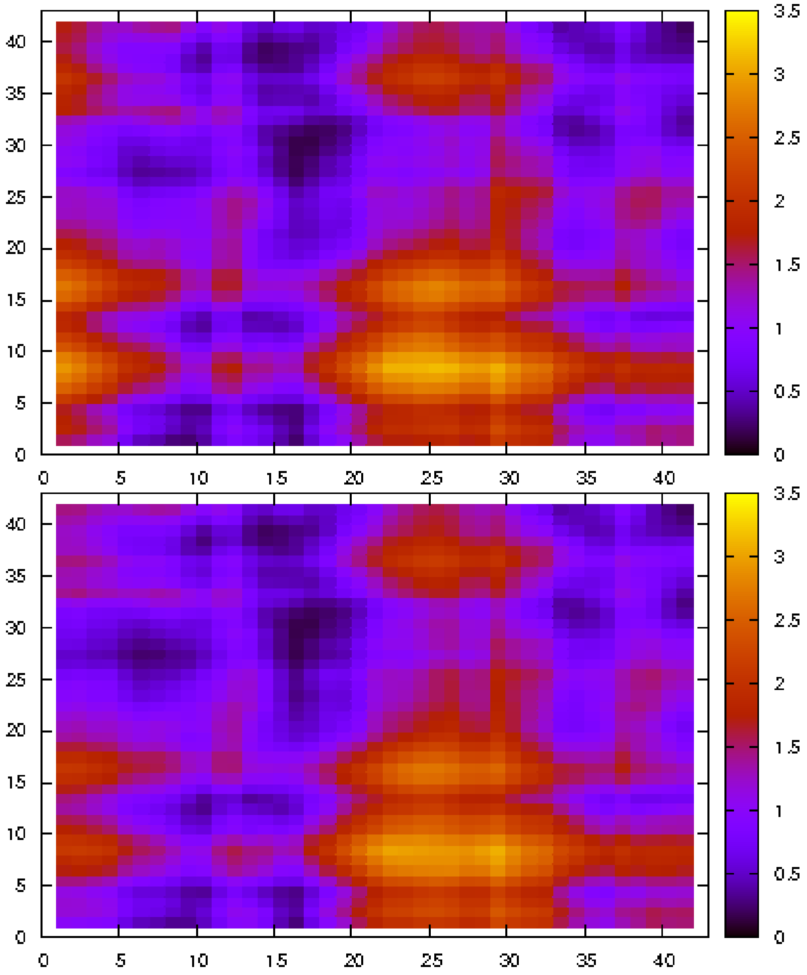

Figure S4. Contacts map between the residues of one protomer and the following one, within the Penta1 (top) and Penta2 (bottom) oligomers. The minimal distances between each pair of residues were averaged over the MD trajectory and over the five protomer-protomer interfaces.

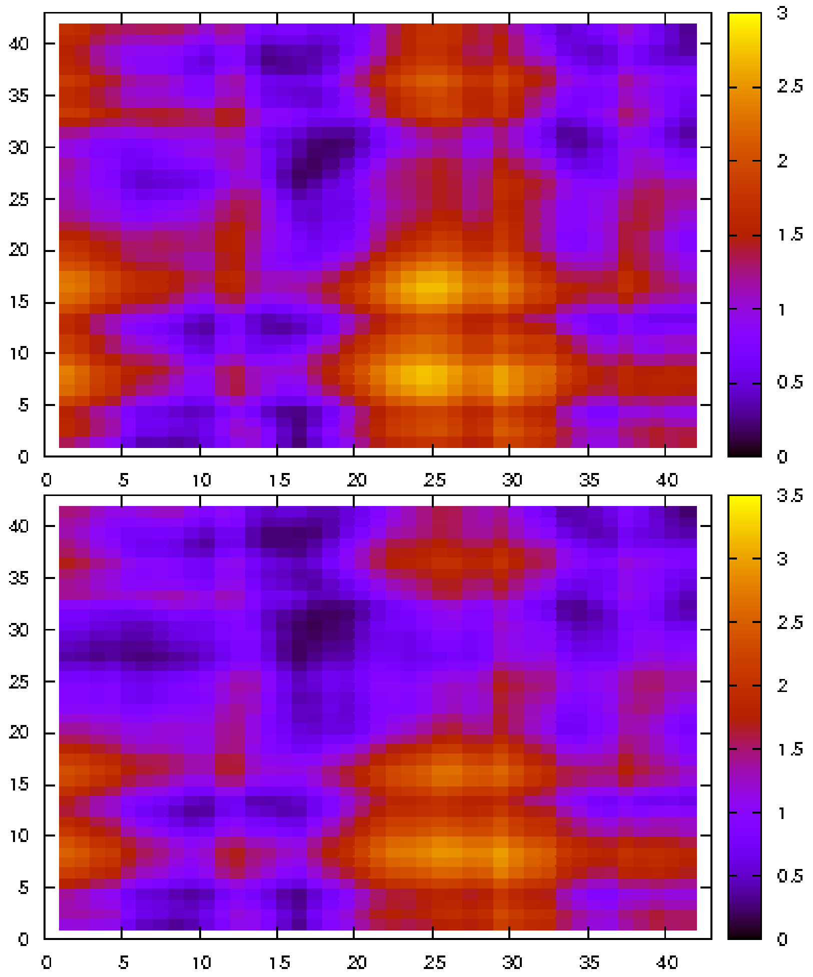

Figure S5. Same as Fig. S4 but for the Hexa1 (top) and Hexa2 (bottom) oligomers.

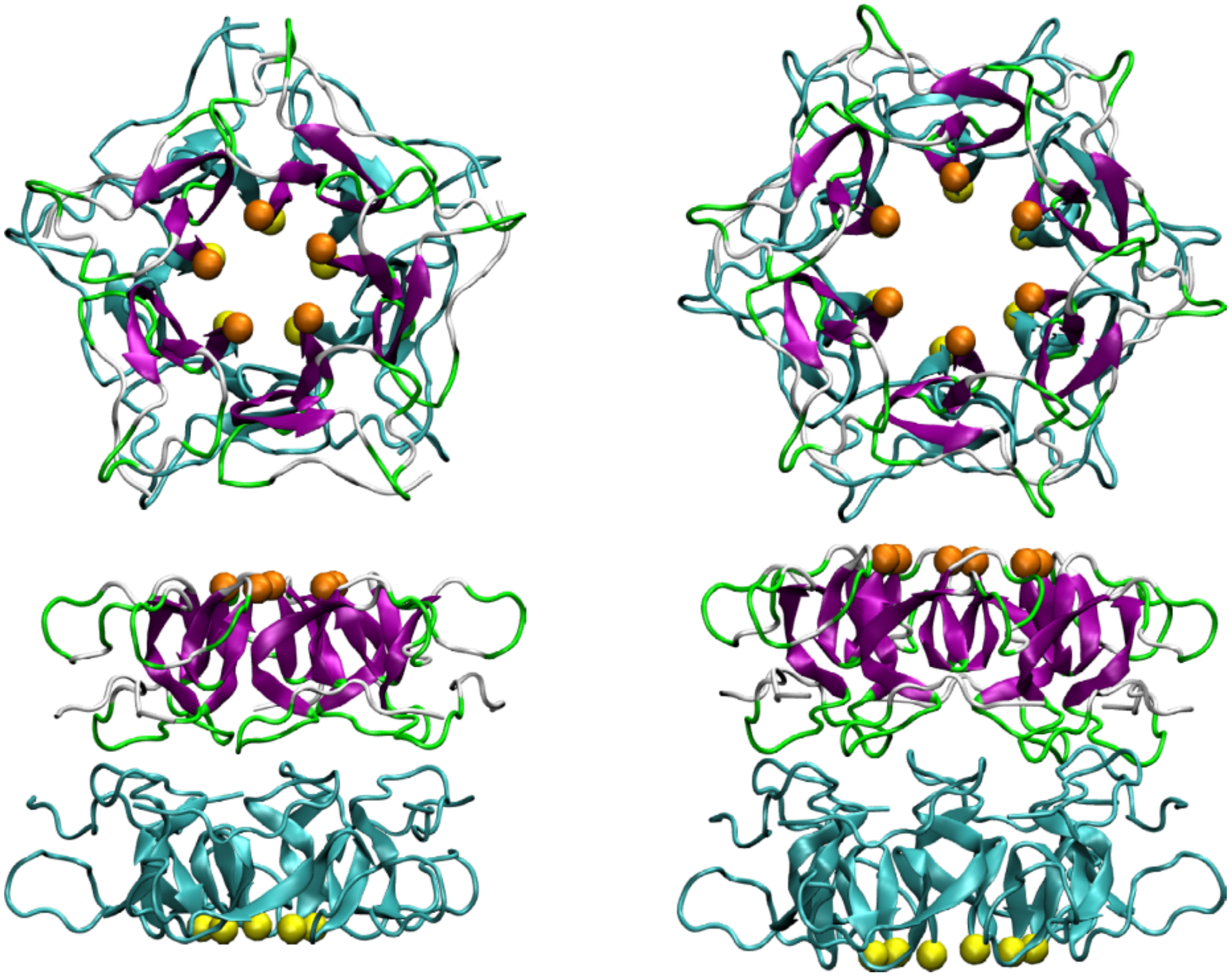

Figure S6. Top and side views of disc-shaped decamer and dodecamer of Aβ_{42} generated by pentamer and hexamer docking calculations on themselves.

References

1. Wälti, M. A., Orts, J., Vögeli, B., Campioni, S. & Riek, R. Solution NMR studies of recombinant Aβ(1-42): From the presence of a micellar entity to residual β-sheet structure in the soluble species. *ChemBioChem* **16**, 659–669 (2015).
